# The Polymorphism rs17525495 of LTA4H Is Associated with Susceptibility of Crohn's Disease instead of Intestinal Tuberculosis in a Chinese Han Population

**DOI:** 10.1155/2019/9537050

**Published:** 2019-04-10

**Authors:** Zi-qi Yu, Wen-fei Wang, Chuan-zhi Zhu, Ke-hong Zhang, Xin-chun Chen, Jian-yong Chen

**Affiliations:** ^1^Department of Gastroenterology & Hepatology, Jiangxi Provincial People's Hospital, Nanchang, China; ^2^Jiangxi Medical College, Nanchang University, Nanchang, China; ^3^Department of Microbiology and Immunology, Shenzhen University Health Science Center, Shenzhen, China; ^4^Department of Pharmaceutical/Medicinal Chemistry, Institute of Pharmacy, Friedrich Schiller University, Jena, Germany; ^5^Laboratory of Molecular Biology, Beijing Chest Hospital, Capital Medical University, Beijing Tuberculosis and Thoracic Tumor Research Institute, Beijing, China

## Abstract

**Background:**

Because of the similarity of intestinal tuberculosis and Crohn's disease in disease phenotype, differential diagnosis has always been a clinical problem. Arachidonic acid metabolites play an important role in the inflammatory response of intestinal tuberculosis and Crohn's disease. Recent studies have shown that the polymorphism locus in the promoter region of* LTA4H* gene affects LTB4 expression level and the susceptibility to extrapulmonary tuberculosis. Thus, we identified a total of 148 patients with intestinal tuberculosis, 145 with Crohn's disease, and 700 normal controls in this study.

**Methods:**

All the study participants were local Han people from Jiangxi Province in the past eleven years. DNA was extracted from the paraffin-embedded specimens or the whole blood. The* LTA4H* promoter SNP (rs17525495) was genotyped with TaqMan assay.

**Results:**

The T-alleles frequency was not significantly increased in patients with intestinal tuberculosis compared with healthy control group (*p*=0.630; OR=1.07; 95%CI=0.81-1.41), while patients with Crohn's disease have significantly increased T allele frequency compared with healthy population (*p*=0.032; OR=1.34; 95%CI=1.03-1.75). During treatment, the presence of the T allele significantly increased the proportion of Crohn's patients requiring glucocorticoids (*p*<0.05).

**Conclusions:**

The T allele of* LTA4H* gene SNP (rs17525495) is a risk factor for Crohn's disease instead of intestinal tuberculosis. More importantly, there may be a potential association of the different genotypes of rs17525495 with the treatment efficacy of 5-ASA and glucocorticoids in patients with Crohn's disease. The association between* LTA4H* polymorphism and drugs therapeutic effects might contribute to the practice of precision medicine and the prediction of clinical outcomes.

## 1. Introduction

Intestinal tuberculosis (ITB) is a chronic granulomatous inflammation caused by M. tuberculosis invading the gut [1]. Crohn's disease (CD) is a chronic idiopathic inflammatory disease of the gastrointestinal tract. Although the exact cause is unknown, it's believed to result from the interplay between genetic susceptibility, environmental factors, and intestinal microflora, showing an abnormal mucosal immune response and compromised epithelial barrier function [2]. Asia was previously considered to be a low-endemic area; however, the incidence has considerably increased over recent decades due to the progress of industrialization and the westernization of lifestyle [3]. On the other hand, globalization and the HIV pandemic have also caused a surge in incidence of TB and consequently abdominal TB in the western world [4, 5]. It has always been a dilemma to distinguish ITB patients from CD patients due to the dynamic changes of epidemiology and the similar characteristics of clinical examination, especially in developing countries [6, 7]. After ITB was misdiagnosed as CD, the immunosuppressive agents treatment may lead to aggravation of tuberculosis infection even systemic dissemination. As for CD misdiagnosed as ITB in reverse, more serious drug side effects and delay treatment will happen after anti-tuberculosis treatment.

Leukotriene A4 hydrolase (LTA4H) is a key enzyme of the 5-lipoxygenase (5-LO) pathway, the activation of which leads to the biosynthesis of proinflammatory leukotriene lipid mediators from arachidonic acid (AA) [8, 9]. The genetic variation of* LTA4H* affects the balance between proinflammatory and anti-inflammatory responses in the body. Therefore, it is closely related to the occurrence and development of various diseases such as asthma, atherosclerosis, infectious diseases, and tumors [10–13]. Through the 1000 Genomes Project, researchers confirmed that rs17525495 genotype affects LTA4H transcription and interferes with therapeutic outcomes of TB meningitis [14]. In a Chinese population study,* LTA4H* gene polymorphism was also reported to be associated with extrapulmonary tuberculosis [15]. In Crohn's disease, James Jupp et al. reported a 4-fold increase in the number of LTA4H-stained cells in colon biopsies from patients with active inflammatory bowel disease compared to healthy controls [16]. However, there is no report on the analysis of the* LTA4H* gene polymorphism in ITB and CD. Hence, in this study, we attempt to genotype the ITB and CD at the rs17525495 locus of the* LTA4H* gene in order to find the difference in genetic susceptibility between ITB and CD.

## 2. Methods

### 2.1. Study Participants

This case-control study was conducted between January 2008 and December 2018 at the Jiangxi Provincial People's Hospital (Nanchang, China) and The First Affiliated Hospital of Nanchang University (Nanchang, China) and The Second Affiliated Hospital of Nanchang University (Nanchang, China). A total of 148 patients with ITB, 145 with CD, and 700 normal controls were enrolled in this study. All of the patients and normal controls were randomly selected from the three hospitals, and all the subjects were local Han people from Jiangxi Province. Inclusion and exclusion criteria for patients with intestinal tuberculosis were the following: age 18 years or older; diagnosed by clinical manifestations, endoscopy, X-ray imaging, pathological changes, positive acid-fast staining, and anti-tuberculosis treatment; no other infectious diseases, chronic diseases, and autoimmune diseases. Inclusion and exclusion criteria for patients with Crohn's disease are the following: age 18 years or older; CD diagnosed based on clinical symptoms, X-ray findings, serological examination results, endoscopic findings, and histopathology following the Chinese Society of Gastroenterology consensus on the diagnosis and management of inflammatory bowel disease, and exclusion of infectious and other noninfectious colitis. The inclusion and exclusion criteria for healthy controls were the following: age 18 years or older; tuberculosis-specific IGRA release test negative; no tuberculosis clinical manifestations (no abnormalities in chest radiology); no other infectious diseases, chronic diseases, and autoimmune diseases. We find out the Paraffin-embedded biopsy specimens of ITB and CD patients and then cut the FFPE sample into 3 sections 20 *μ*m thick and transfer into EP tube stored at −20°C immediately. 2 ml of lithium heparin anticoagulated whole blood was collected from the healthy controls, at 4°C for storage. The present study protocol was reviewed and approved by the institutional review board of the above three hospitals and complied with the principles of the Declaration of Helsinki.

### 2.2. Genotyping for* LTA4H* Gene rs17525495 Locus Polymorphisms

We used FFPE DNA Kit (OMERA, US) for extraction of genomic DNA from paraffin-embedded specimens of ITB and CD patients as described above. We also used DNeasy Blood & Tissue (QIAGEN, US) for extraction of genomic DNA from whole blood in healthy controls (HC). The SNP rs17525495 was genotyped with TaqMan assays in 20 *μ*l reaction volume, using ABI 7500 real-time PCR systems (ABI, US). Cycling conditions were set up as follows: pre-PCR Read (Holding Stage) 60°C for 1 min and then Holding Stage 95°C for 10 min, followed by 40 cycles at 95°C for 15 s, 60°C for 1 min, and Post-PCR Read (Holding Stage) 60°C for 1 min. Results were analyzed by using ABI 7500 PCR instrument software.

### 2.3. Statistical Analysis

In this case-control study, we first compared the baseline characteristics of study population. The categorical variables were analyzed by chi-square test or Fisher's exact test, and the continuous variables were compared using independent sample t-test. The goodness-of-fit *χ*^2^ test was performed to check if the genotype frequencies of case and control groups conformed to Hardy-Weinberg equilibrium. Power calculations were performed using Power and Sample Size Calculation Statistical Software [17]. Unconditional logistic regression analysis was used to compare the genotype and allele frequencies among the three groups. Covariates as gender, age, smoking, and drinking were included to adjust the odds ratio (OR) value. The disease-associated Additive genetic model was evaluated by the Cochran-Armitage trend test. For the disease with significant results in the additive model text, logistic regression analysis was used to analyze the three genetic models (dominant, recessive, and codominant) that the rs17525495 locus may follow in the disease; OR and 95% confidence interval (CI) were calculated and then adjusted by using the above covariates. All statistical analyses were performed by using SPSS (statistical software version 24). The value of* p*<0.05 (two-sided) was considered statistically significant.

## 3. Results

### 3.1. Clinical Characteristics

The clinical characteristics of study population are shown in Table 1. No significant differences were found between the ITB and CD groups in terms of gender, smoking and drinking history, WBC, Hb, ESR, PPD, or disease related surgery (*p*>0.05). However, the average diagnosis age of patients with CD is earlier than ITB, and both of T-SPOT positive rate and abnormal lung imaging findings showed significant differences between the two groups (*p*<0.05), indicating their essential role in identifying ITB and CD.

### 3.2. SNP Distributions

The genotype distributions of rs17525495 in the case and control groups conformed to Hardy-Weinberg equilibrium (*p*>0.05). Retrieving rs17525495 gene in the dbSNP database, we found that the MAF (Minor Allele Frequency) was 0.29 in the East Asian population (China & Japan), and the statistical power of the polymorphism study exceeded 0.75. The genotype and allele frequency distribution of rs17525495 and its relationship with ITB and CD in Chinese Han population were shown in Table 2. After adjusting for age, gender, and other covariates, there was no significant difference in genotype and allele frequency regarding* LTA4H* rs17525495 when we compared with HC and ITB, or ITB and CD. However, a comparison between patients and controls showed a significant association between the TT genotype and Crohn's disease (*p*=0.034; OR=1.92; 95%CI=1.05-3.51); the presence of the T allele might function as one of the genetic risk factors for Crohn's disease (*p*=0.032; OR=1.34; 95%CI=1.03-1.75).

### 3.3. Genetic Model Analysis

In the additive model of ITB (*χ*^2^=1.07;* p*=0.30) and CD (*χ*^2^=5.98;* p*=0.01), there was no significant correlation between the genotype of LTA4H rs17525495 locus and ITB susceptibility, whereas there was significant association with CD. In addition, a significant difference was found between the wild-type and mutant homozygotes under the codominant model, and the difference was still statistically significant after adjusting for the covariates (*p*=0.035; OR=1.94; 95%CI=1.05-3.57). The results were shown in Table 3. It suggests that this site may follow a codominant model in the CD.

### 3.4. The Association of Hematological Examination and Genotype

There were no statistical differences among the three clinical indicators (WBC, Hb, and ESR) in patients with different genotypes of rs17525495 in both ITB and CD patients. The result was shown in Supplementary Figure 1 (Figure S1).

### 3.5. The Association of Medicinal Cure Effect and Genotype in CD Patients

**Figure 1:**
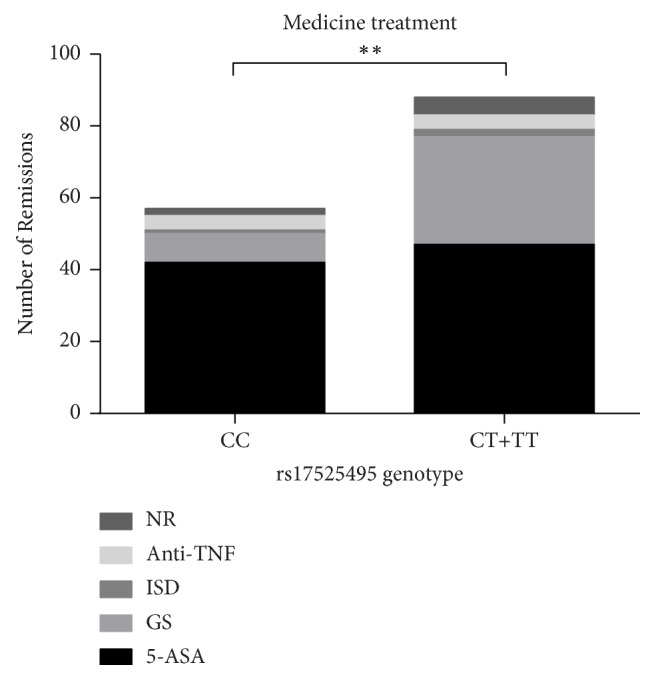
The association of medicinal cure effect and genotype in CD patients. 5-ASA: 5-Aminosalicylic acid; GS: glucocorticoids; Anti-TNF; Infliximab, IFX; ISD: immunosuppressant, Azathioprine; NR: nonremission. In the early stage of CD treatment, the least toxic drugs are preferred. If they are ineffective or have adverse reactions, it will switch to other treatment options. The usual treatment starts with 5-Aminosalicylic acid and gradually advances to the immunosuppressant via glucocorticoids and biological agents in the end. Clinical response or remission was used to assess the efficiency of the drugs. We classify CD patients by genotype. *∗∗* Adjusted for gender and age.

Among CD patients, we compared TT+CT with CC genotype to see if genotypes affect the efficacy of drug therapy (Figure 1). The presence of the T allele significantly increased the proportion of Crohn's patients with requiring GS (*p*<0.05).

## 4. Discussion

The development and maintenance of inflammation are controlled by a complex network of cells and cytokines. Among these are the eicosanoids: a class of structurally related paracrine hormones, derived from the metabolism of arachidonic acid that includes the prostaglandins, the leukotrienes, and lipoxins, play an important role in the inflammatory response [18]. In the 5-LO pathway, LTA4H catalyzes the conversion of LT4H to LTB4 [19]. LTB4 is a potent lipid chemoattractant involved in inflammation, which causes adhesion and chemotactic movement of inflammatory cells and induces macrophage release of oxygen radicals [20], which is also reported to stimulate the production of IL-6 and IFN-*γ* and TNF [21–23]. In addition, lipoxygenase-derived metabolites of arachidonic acid may also be involved in activation, proliferation, and differentiation of human lymphocytes [24, 25]. For several reasons, the expression level of LTA4H plays an important role in the body's inflammatory response.

Rs17525495 is one of the tag SNPs of* LTA4H*, which is located in the promoter region of the gene. David M. Tobin et al. confirmed protein expression of rs17525495 TT group shows approximately 2.3-fold higher than CC group, with intermediate expression in the CT cell lines, and the locus affects transcriptional activity through conducting dual-luciferase assays [14]. Therefore, rs17525495 is a functional SNP. This locus was originally studied in tuberculosis; among TB meningitis infected patients without dexamethasone treatment, the patients with TT genotype had the highest mortality. In dexamethasone treated group, the result is in reverse [14, 26]. Interestingly, SNP heterozygosity in tuberculosis infection showed a protective effect, whereas both homozygous appeared to be increasing disease severity. The reason might be inadequate or excessive acute inflammation influences the susceptibility to M. tuberculosis [14, 26, 27].

We found that the frequency of T allele showed no significant difference between ITB group and control. This is different from the findings of tuberculous meningitis in Vietnamese population [28]. This may be related to the following three reasons. Firstly, there were differential effects of genetic polymorphisms on susceptibility to disease in various populations. In a large sample of Russian population research, the results suggest that the other six common polymorphisms in the* LTA4H* gene do not play any major role in susceptibility to clinical pulmonary tuberculosis [29]. Secondly, related reports on the impact of this locus on susceptibility to tuberculosis are mainly in pulmonary tuberculosis and tuberculous meningitis. As we know, M. tuberculosis genotype influences clinical disease phenotype. More virulent Beijing/W lineage strains are associated with tuberculous meningitis [30]. However, some of the infections in ITB are caused by the ingestion of dairy products contaminated with Mycobacterium bovis, which are less virulent mycobacteria [31]. Furthermore, because the sample of ITB is relatively small, the possibility of significant differences after expanding the sample size cannot be ruled out.

The contribution of AA metabolites pathway in CD has been recognized [32]. These metabolites are important in promoting the migration of neutrophils to the intestinal mucosa, which cause substantial epithelial damage by producing reactive oxygen species and proteases [33]. Previous studies have shown that mucosal synthesis of LTB4 is greatly enhanced in CD. In particular, the increased, sustained, and recurrent inflammation characteristic of CD may be the result of an inherent defect in the metabolism of LTB4, leading to its enhanced accumulation and activity [34]. The classical anti-inflammatory drugs for CD treatment such as 5-aminosalicylic acid and glucocorticoids are mainly through inhibition of PGE2 and LTB4 release in colonic mucosa to reduce inflammation [35]. A Canadian study evaluated the proteomes of biopsies collected from 99 pediatric patients at diagnosis-stage and discovered and validated the potential protein biomarkers through quantifying over 3500 proteins. They found proteins involved in fatty acid metabolism to be elevated in the CD over UC pediatric patients, especially the protein LTA4H [36]. Another research suggested that LTA4H is a special biomarker of CD diagnosis and severity evaluation. Whatever in intestinal mucosal-luminal interface (MLI) or stool samples, higher protein expression of LTA4H is related to CD [37]. We hypothesize that patients with high expression of LTA4H produce more LTB4, causing an excessive acute inflammatory response, and thus glucocorticoids can effectively inhibit the progression of inflammation and improve the symptoms of patients.

## 5. Conclusion

In conclusion, we first report that the* LTA4H* SNP rs17525495 T allele is a risk factor for CD. More importantly, there is a clear correlation between genotype and drug efficacy. Further research on the interaction of* LTA4H* SNPs will provide a theoretical background for elucidating the role of arachidonic acid metabolites in tuberculosis and inflammatory bowel disease and may help the susceptible population to take host genotype-specific therapies earlier to achieve the best curative effect.

## Figures and Tables

**Table 1:** Baseline characteristics of study population.

	HC (n=700)	ITB (n=148)	CD (n=145)	*∗*P-value
Gender (Female/Male)	332/368	48/100	47/98	0.80
Age of diagnosis (years)	38.5 ± 12.8	41.7 ± 16.7	36.6 ± 15.2	***0.008***
Smoker, n (%)	144/700(20.6)	35/148 (23.6)	27/145 (18.6)	0.29
Drinker, n (%)	79/700(11.3)	19/148 (12.8)	18/145 (12.4)	0.91
WBC (10^∧^9/L)		7.15 ± 3.9	7.72 ± 3.4	0.30
Hb, g/L		110.20 ± 24.2	108.37 ± 25.5	0.56
ESR, mm/h		38.15 ± 24.8	35.20 ± 23.9	0.47
PPD (positive), n (%)		38/56 (67.9)	15/43 (34.9)	0.07
T-SPOT (positive), n (%)		25/33 (75.8)	3/31 (9.68)	***0.0006***
Not examined TB		59	71	
Abnormal lung imaging findings, n (%)		56/148 (37.8)	4/145 (2.8)	***< 0.0001***
Remission rate: n (%)				
5-ASA			88/145 (60.7)	
Glucocorticoid			34/145 (23.4)	
Immunosuppressor			7/145 (4.8)	
Anti-TNF			9/145 (6.2)	
Nonremission			7/145 (4.8)	
Disease related surgery, n (%)		23/148 (15.5)	33/145 (22.8)	0.20
Location of ITB, n (%)				
Small Intestine		12/148 (8.1)		
Ileocecus		89/148 (60.1)		
Colon		39/148 (26.4)		
Data not available		8/148 (6.0)		
Location of CD, n (%)				
L1± L4			67/145 (46.2)	
L2± L4			41/145 (28.3)	
L3± L4			34/145 (23.4)	
L4			3/145 (2.0)	
Behavior of CD, n (%)				
B1—Inflammatory			95/145 (65.5)	
B2—Stricturing			45/145 (31.0)	
B3—Penetrating			10/145 (6.9)	
p—Perianal			37/145 (25.5)	

Smoker: subjects who had smoked more than one cigarette per day for at least one year; drinker: subjects who drank alcohol per week for more than six months; ESR: erythrocyte sedimentation rate; PPD, tuberculin skin testing. Crohn's disease phenotype defined by the Montreal Classification System, L1, Terminal ileum; L2, colonic; L3, ileocolonic; L4, upper GI. *∗* P-value means comparison between ITB and CD groups. Bold italics indicate the statistical significance (*P* < 0.05).

**(a):** 

Genotype & Allele	HC	ITB	OR (95% CI)	*P*	OR (95% CI)^*∗*^	*P* ^*∗*^
CC	336	72	1.00		1.00	
CT	311	58	0.87(0.60-1.27)	0.472	0.88(0.60-1.28)	0.495
TT	53	18	1.59(0.88-2.87)	0.127	1.44(0.79-2.63)	0.234
C	983	202	1.00		1.00	
T	417	94	1.10(0.84-1.44)	0.502	1.07(0.81-1.41)	0.630

**(b):** 

Genotype & Allele	HC	CD	OR (95% CI)	*P*	OR (95% CI)^*∗*^	*P* ^*∗*^
CC	336	57	1.00		1.00	
CT	311	69	1.31(0.89-1.92)	0.170	1.31(0.89-1.92)	0.178
TT	53	19	2.11(1.17-3.83)	***0.014***	1.92(1.05-3.51)	***0.034***
C	983	183	1.00		1.00	
T	417	107	1.38(1.06-1.80)	***0.017***	1.34(1.03-1.75)	***0.032***

**(c):** 

Genotype & Allele	ITB	CD	OR (95% CI)	*P*	OR (95% CI)^*∗*^	*P* ^*∗*^
CC	72	57	1.00		1.00	
CT	58	69	1.50(0.92-2.50)	0.105	1.53(0.93-2.53)	0.094
TT	18	19	1.33(0.64-2.77)	0.441	1.26(0.60-2.66)	0.539
C	202	183	1.00		1.00	
T	94	107	1.26(0.89-1.77)	0.190	1.24(0.88-1.75)	0.226

The major genotype or allele was used as baseline.**∗** Adjusted for gender, age, smoking, and drinking history. Bold italics indicate the statistical significance (*P* < 0.05).

**Table 3:** Genetic model analysis of rs17525495 in CD population.

Genetic model	Genotype	HC (n)	CD (n)	OR (95% CI)	*P*	OR (95% CI)^*∗*^	*P* ^*∗*^
Dominant	CC	336	57	1.00		1.00	
	CT+TT	364	88	1.43 (0.99-2.05)	0.057	1.40 (0.97-2.03)	0.074

Recessive	CT+CC	647	126	1.00		1.00	
	TT	53	19	1.84 (1.05-3.22)	***0.032***	1.68 (0.95-2.95)	0.074

Codominant	CC	336	57	1.00		1.00	
	TT	53	19	2.11 (1.17-3.83)	***0.014***	1.94 (1.05-3.57)	***0.035***
	CC	336	57	1.00		1.00	
	TC	311	69	1.31 (0.89-1.92)	0.17	1.30 (0.88-1.92)	0.182

The major genotype or allele was used as baseline.**∗** Adjusted for gender, age, smoking, and drinking history. Bold italics indicate the statistical significance (*P* < 0.05).

## Data Availability

The data used to support the findings of this study are included within the article.

## Supplementary Materials

Supplementary Materials Figure S1: the association of hematological examination and genotype.
