# Supplementary material for: The Polymorphism rs17525495 of LTA4H Is Associated with Susceptibility of Crohn's Disease instead of Intestinal Tuberculosis in a Chinese Han Population
Source: Biomed Res Int. 2019 Apr 10;2019:9537050. doi: 10.1155/2019/9537050 (PMC6481108; doi:10.1155/2019/9537050)
Supplement: Supplementary Materials — Figure S1: the association of hematological examination and genotype. [file 9537050.f1.docx]

**Figure S1**: The association of hematological examination and genotype

**
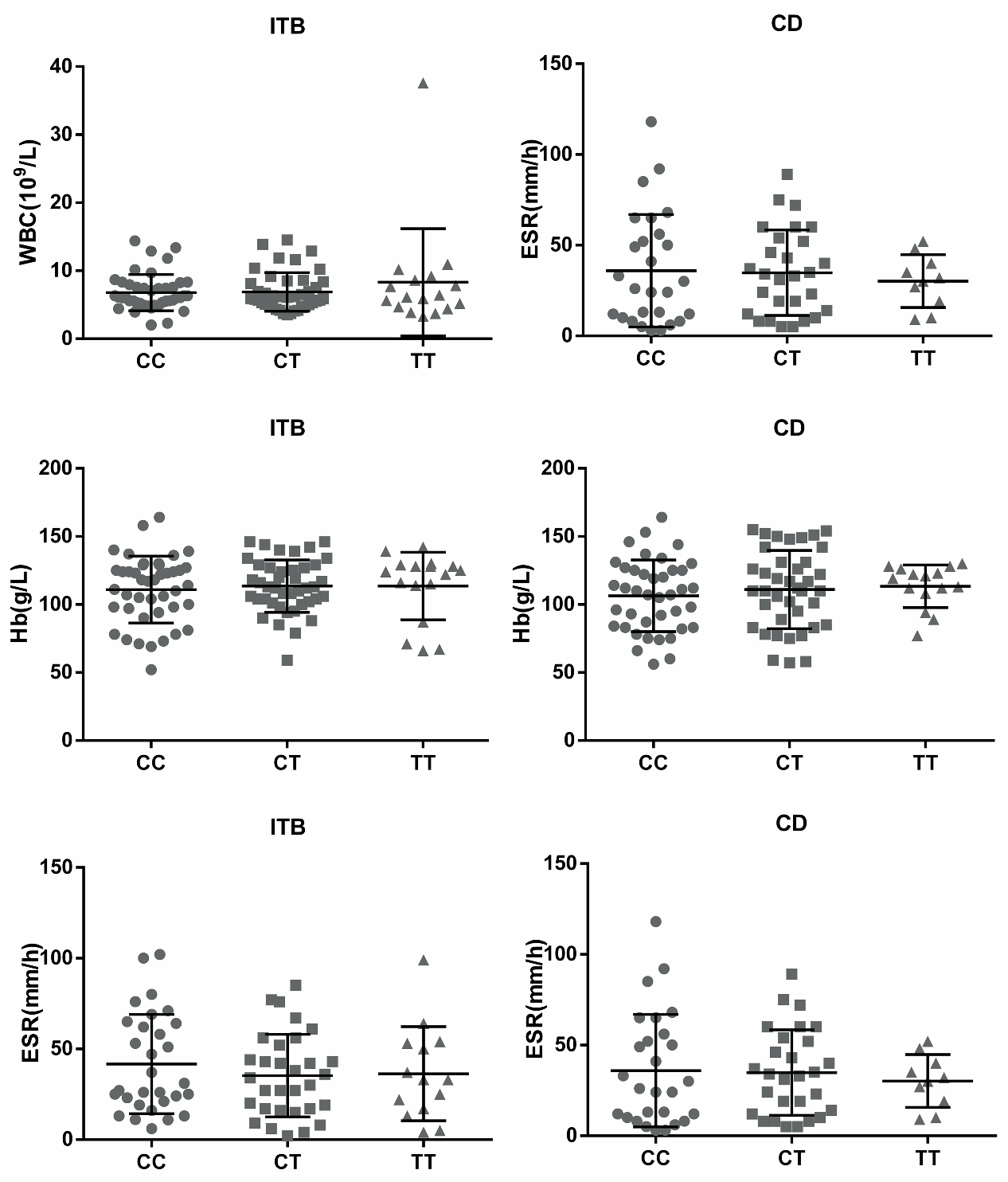
**

WBC: White blood cell count; Hb: Hemoglobin; ESR, Erythrocyte Sedimentation Rate. Some of the data, ESR in particular, is missing because the patients are not being examined.
